# Cyclodextrins: Properties and Applications

**DOI:** 10.3390/ijms25084547

**Published:** 2024-04-21

**Authors:** Miguel A. Esteso, Carmen M. Romero

**Affiliations:** 1 Faculty of Health Sciences, Universidad Católica de Ávila, Calle los Canteros s/n, 05005 Ávila, Spain; 2 U.D. Química Física, Universidad de Alcalá, 28805 Alcalá de Henares, Spain; 3 Departamento de Química, Facultad de Ciencias, Universidad Nacional de Colombia, Sede Bogotá, Carrera 30 No. 45-03, Bogotá 111311, Colombia

## 1. Introduction

Cyclodextrins (CDs) are cyclic oligosaccharides that contain at least six d–(+)–glucopyranose units linked by α–(1, 4) glucosidic bonds. The three natural CDs, α-, β-, and γ-CDs, have 6, 7, and 8 glucose units, respectively, and differ from each other in their size and solubility. CDs with less than six glucose units cannot form due to steric hindrance, and CDs with nine or more glucose units are difficult to purify.

In 1891, Antoine Villiers discovered crystalline dextrins and was the first to isolate the oligosaccharides produced by starch or its derivatives using the cyclodextrin (CD) glycosyltransferase enzyme. The structure of CDs was described 30 years later by Freudenberg and his co-workers, and they were produced in pure form in 1984. Since then, numerous scientific publications and patents have been developed focusing on their diverse applications in the pharmaceutical and food industries, products, and technologies [1,2]. The most common natural CDs used in industrial-scale production are alpha-cyclodextrin (α–CD), beta-cyclodextrin (β–CD), and gamma-cyclodextrin (γ–CD) [1,2]. 

Cyclodextrins belong to the family of cage-type molecules. They have a non-symmetrical toroidal structure, wider at one end and narrower at the other in a truncated cone shape. They have a hydrophilic exterior, hydroxyl groups at both ends, and a hydrophobic inner cavity. Due to their shape, CDs can encapsulate other molecules in aqueous solutions, allowing a wide range of hydrophobic guest molecules to interact with the inner cavity of the macrocycle to form inclusion complexes. Cyclodextrins’ encapsulation capacity and flexibility allow host−guest-type interactions to modify the physical, chemical, and biological properties of the guest molecules [1,2,3,4,5].

Cyclodextrins continue to be of interest to many researchers, due to their encapsulation capacity and their properties as complexing agents and as carriers of different substances, which make them good candidates for both fundamental and technological applications in several industries. For this reason, important experimental [1,2,3,4,5] and theoretical studies [6,7] have been performed, focused on their physicochemical behavior and applications.

Due to their biocompatibility, biodegradability, and relatively low-cost production [8,9,10], cyclodextrins are widely used in several industries, such as the medical, pharmaceutical, cosmetics [11,12,13,14], food [4,15,16,17], and textile industries [18], and in different processes related to biotechnology, agriculture, and the environment. Likewise, they are used in controlled release systems, to improve the solubility and bioavailability of poorly water-soluble substances, and as catalysts for different reactions [19,20].

Despite their theoretical and applied importance, and the extensive literature available, little is known about the significant potential of these molecules, which necessitates research into the structure and properties of these macrocycles [21,22]. In this sense, a promising line of study is modifying standard CDs’ structure and synthesis to obtain new ones with greater complexing capacity toward different substances, either as a consequence of their inclusion within the cavity of the CD or their bonding to the external part. Another important area of study is the physicochemical properties of the aqueous solutions of these systems for the elucidation of the nature of host−guest and inclusion complex−aqueous solvent interactions. In particular, the solubilizing capacity of CDs is of great practical importance. Finally, research on the novel applications of these important molecules to address environmental problems, synthesize new nanoparticles, and construct nano-devices for medical applications, among other applications, will reinforce their potential use [1,2,3,4,5,22,23].

Although cyclodextrins are considered toxic, their toxic effects can be eliminated by selecting the appropriate CD type, using derived compounds with harmful properties removed, and selecting appropriate concentrations and modes of application. Once the toxic effects are removed, CDs can be consumed as ingredients in drugs, foods, or cosmetics [22].

## 2. An Overview of Published Articles

This Special Issue, *Cyclodextrins: Properties and Applications*, highlights research papers or comprehensive reviews that focus on advances in the knowledge of the structure, properties, and applications of cyclodextrins (CDs). It provides a comprehensive overview of current research related to these compounds. Fourteen papers were accepted for publication and inclusion in this Special Issue. Topics include design, synthesis, characterization, and other applications. See list of contributions.

Contribution 1 develops eye drop formulations using voriconazole (VCZ), cyclodextrins (CDs), and water-soluble polymers, forming CD complex aggregates to improve VCZ’s solubility in water and chemical stability. Among the CDs studied, SBE–β–CD was the most effective CD solubilizer of VCZ/SBE–β–CD complexes, and the VCZ solubility was greatly enhanced using sulfobutyl ether β–cyclodextrin (SBE–β–CD). Polyvinyl alcohol (PVA) promotes the solubilization and stabilization of the VCZ/SBE–β–CD complex and less SBE–β–CD is required, which may be advantageous in terms of toxicology and manufacturing costs.

Contribution 2 analyzes the interaction between sodium salicylate (NaSal) and the two macrocycles 5,11,17,23–tetrakissulfonatomethylene–2,8,14,20–tetra(ethyl)resorcin arene (Na_4_EtRA) and β–cyclodextrin (β–CD) using spectroscopic and computational techniques, as well as by determining mutual diffusion coefficients. Both systems show the formation of 1:1 host–guest complexes. For the β–CD–NaSal complex, the calculated diffusion coefficients and complexation energies indicate that an inclusion complex occurs, while when Na_4_EtRA–NaSal is used, an inclusion complex is not formed.

Contribution 3 deals with the interaction between β–cyclodextrin (β–CD) and melphalan (Mel), which is an antineoplastic widely used to treat cancer and other diseases. The 1:1 solid crystalline complex formed prevents the hydrolysis and subsequent degradation of the drug and is a useful tool in drug delivery for cancer. 

Contribution 4 is related to the use of a supramolecular complex of large-ring cyclodextrins (LR–CDs) and α–tocopherol, the most physiologically active form of vitamin E with numerous biological activities, such as significant antioxidant activity, anticancer capabilities, and anti-aging properties. The results show that the most probable ratio of the inclusion complex is 2:1 and the interaction improves the solubility and bioavailability of α–tocopherol in pharmaceutical applications.

Contribution 5 describes a fluorescence method for analyzing the nucleocapsid protein (N protein), which is an appropriate target for the early diagnosis of severe acute respiratory syndrome coronavirus 2 (SARS-CoV-2) based on viral antigens. The method combines the host–guest interaction fluorescence enhancement strategy with the high recognition of aptamers. The outstanding fluorescence enhancement during the host–guest interaction between β–CDP and pyrene and the mutually repulsive force between the negatively charged aptamer and β–CDP ensured low background noise and had high sensitivity.

Contribution 6 focuses on the preparation and biological, physiochemical, and theoretical analysis of the inclusion complexes formed between estrogens and cyclodextrins (CDs). Estrogens have a low polarity and can interact with cyclodextrins’ hydrophobic cavities to create inclusion complexes. The paper focuses on the effect of CDs as estrogen solubilizers and absorption boosters in pharmaceutical formulations, as well as on chromatographic and electrophoretic procedures for their separation and quantification. The review shows that the cyclodextrin type used for complexation can greatly influence the properties of the complex formed, such as the dissolution boost of the guest molecule, the host−guest ratio, or the complex stability.

Contribution 7 describes the obtention of three novel biomaterials via inclusion complexes of β–cyclodextrin, 6–deoxi–6–amino–β–cyclodextrin, and epithelial growth factor grafted on 6–deoxi–6–amino–β–cyclodextrin with polycaprolactone. The in silico study of these three novel materials was based on quantum chemistry and chemoinformatics methods. The results contribute to the theoretical characterization of biomaterial derivatives of β–cyclodextrin inclusion complexes.

Contribution 8 determines the most suitable cyclodextrins (CDs) for solubilizing a patented succinimido−ferrocidiphenol (SuccFerr), a compound from the ferrociphenol family with powerful anticancer activity but low water solubility. Modeling and phase solubility experiments suggest the predominance of supramolecular SuccFerr assemblies with two CDs and the superiority of randomly methylated β–cyclodextrins (RAME–β–CDs). According to the authors, the method allows the modeling of the atypical hydrogen bonds between CDs and a nonpolar ferrocene-based anticancer molecule.

Contribution 9 prepares inclusion complexes of β– and γ–cyclodextrin with enzymatic hydrolysates of whey and colostrum proteins. The effect of cyclodextrin complexation on the antioxidant properties, antigenic potential, and antimutagenic effect of the included dairy peptides was determined. An increase in the antioxidant effect of hydrolysates in host–guest systems with β– and γ–cyclodextrin was observed. Inclusion complexes of β– and γ–cyclodextrin with whey and colostrum peptides have confirmed bioactive action, making them promising ingredients for functional foods.

Contribution 10 explores complexation with cyclodextrin (CD) as a strategy for overcoming the pharmaceutical limitations, such as unpleasant taste or poor aqueous solubility, that prevent the evaluation and clinical use of 4–phenylbutyrate (PB) and structurally related compounds in many diseases, including cancers. The results show that α–CD is more suitable for overcoming the pharmaceutical drawbacks of PB and its shorter-chain derivatives, while β–CD is better for the longer-chain derivatives.

Contribution 11 evaluates FA–HP–β–CD as a novel treatment for acute myeloid leukemia (AML), both in vitro and in vivo. The authors developed folate-conjugated HP–β–CD (FA–HP–β–CD) and investigated its effects on folate receptor (FR)-expressing AML cells. According to the authors, the cytotoxic activity of (FA–HP–β–CD) against AML cells was stronger than that of HP–β–CD. They confirm that FA–HP–β–CD increased the inhibitory effects of cytarabine and the BCL–2-selective inhibitor, Venetoclax, which are commonly used to treat elderly patients with AML. The results show that FA–HP–β–CD induces cell death through a non-apoptotic pathway, making it a new therapeutic option for AML chemotherapy by regulating autophagy.

Contribution 12 explores the conformational potential energy surface of systems with conformational flexibility, such as the monomers and dimers of α–, β–, and γ–cyclodextrins (i.e., of 6, 7, and 8 monomeric units, respectively), using combined semiempirical methods and DFT computational approaches refined by state-of-the-art DFT functions. Moreover, the crystal structure is compared with the experimentally recorded IR spectrum. The authors present a cost-effective methodology to explore the conformational complexity of flexible systems and properly refine the results with a cheap but accurate DFT method applicable to medium-sized systems.

Contribution 13 describes the first silica–CD hybrid through one-pot sol–gel synthesis starting from methyl–β–cyclodextrin (bM–CD) and tetramethyl orthosilicate (TMOS) as a silica precursor. In certain cases, a high specific surface area is desirable for increasing the release/adsorption properties, so the dense hybrid material obtained was prepared as an aerogel. Each hybrid can be used in different applications depending on its properties.

Contribution 14 explores the development of advanced drug delivery systems composed of poloxamer 407, a non-ionic surfactant (Tween 80), and cyclodextrins (methyl–β–CD or hydroxypropyl–β–CD) for treating Parkinson’s disease by the potential brain targeting of ropinirole after nasal administration. The results suggest that hybrid systems combining a polymer, a surfactant, and CDs present promising opportunities for nose-to-brain RH delivery in PAD.
